# Cryopreservation duration does not affect pregnancy or neonatal outcomes in single high-quality blastocyst transfers: a multicenter retrospective study

**DOI:** 10.3389/fendo.2026.1743765

**Published:** 2026-03-06

**Authors:** Tingting He, Xia Xue, Wei Li, Bo Huang, Xinling Ren, Bingxin Ma, Youzhu Li, Lei Jin, Juanzi Shi

**Affiliations:** 1 Assisted Reproduction Center, Northwest Women’s and Children’s Hospital, Xi’an, China; 2 Reproductive Medicine Center, Tongji Hospital, Tongji Medical College, Huazhong University of Science and Technology, Wuhan, China; 3 The Department of Reproductive Medicine, The First Affiliated Hospital of Xiamen University, Xiamen, China

**Keywords:** cryopreservation duration, frozen embryo transfer, high-quality blastocyst, neonatal outcome, pregnancy outcome

## Abstract

**Objective:**

To study the effect of embryo cryopreservation duration on pregnancy and neonatal outcomes in women transferred with high-quality blastocyst during frozen embryo transfer (FET) cycles.

**Design:**

Multicenter Retrospective cohort study.

**Setting:**

Three tertiary-care academic medical centers.

**Patients:**

This retrospective study included a total of 24,101 women who underwent single high-quality blastocyst transfer during their first FET cycles at three tertiary academic medical centers between January 2016 and June 2023.

**Intervention(s):**

Women were categorized into two groups according to the duration of embryo cryopreservation: the short Cryo group consisted of 23,933 women with a storage time of 0–5 years, while the long Cryo group included 168 women with a storage time > 5 years. Women in the long Cryo group were matched to those in the short Cryo group using propensity score matching with a 1:4 ratio.

**Main outcome measure(s):**

The pregnancy outcomes and the neonatal outcomes.

**Result(s):**

After adjusting for potential confounding factors, no significant differences were observed between the two groups in pregnancy outcomes, including biochemical pregnancy (adjust odds ratio [aOR] 1.04, 95% confidence interval [CI], 0.70–1.56; P = 0.831), clinical pregnancy (aOR 1.10, 95% CI, 0.75–1.60; P = 0.638), ectopic pregnancy (aOR 2.15, 95% CI, 0.37–12.56; P = 0.394), miscarriage (aOR 0.95, 95% CI, 0.54–1.69; P = 0.871), and live birth (aOR 1.09, 95% CI, 0.76–1.55; P = 0.646). In addition, no significant differences were observed in neonatal outcomes, including very preterm birth, preterm birth, very low birth weight, low birth weight, high birth weight, birth weight, and gestational age.

**Conclusion(s):**

Our analysis found no evidence of significant associations between prolonged cryopreservation of high-quality blastocysts and adverse pregnancy or neonatal outcomes.

## 1 Introduction

Embryo cryopreservation is a technology to keep embryos in an ultra-low temperature environment (liquid nitrogen, -196 °C) for preservation, and subsequently thawing them to normal physiological temperatures when needed. Since the first successful clinical pregnancy following cryopreserved embryo transfer was reported in 1983, cryopreservation techniques have developed rapidly and become a routine practice in assisted reproductive technology (ART) (1). The use of this technology not only minimizes the waste of embryos but also significantly reduces the risk of ovarian hyperstimulation syndrome (OHSS) (2). Furthermore, it can apply to couples who are unsuitable for fresh embryo transfer either due to uterine receptivity or preimplantation genetic testing (PGT) (3, 4). Additionally, it plays a critical role in fertility preservation, particularly for women diagnosed with cancer or diminished ovarian reserve (4). With the improvement in cryopreservation technology, as well as the adoption of single embryo transfer strategy, the number of frozen embryos and the duration of embryo cryopreservation have been steadily increasing (5, 6).

Traditionally, two main methods have been used to freeze embryos: slow freezing and vitrification (7). In slow freezing, embryos are cooled at a slow rate, facilitating full cellular dehydration and effectively preventing intracellular ice formation. In contrast, vitrification involves rapid cooling, during which embryos undergo full dehydration without the formation of ice crystals, thereby significantly reducing the likelihood of cryodamage (7). Compared with slow freeing, vitrification is considered the most reliable technique in clinical practice for higher survival rate, better clinical outcomes, and notable advantages including simplicity, convenience, and cost-efficiency (8). The widespread use of embryo cryopreservation has led to prolonged duration of embryo preservation prior to transfer. This extended preservation period has raised critical safety concerns, particularly regarding potential osmotic toxicity induced by high concentration cryoprotectants and the risk of contamination associated with prolonged exposure to liquid nitrogen (9).

The impact of cryopreservation duration on reproductive outcomes remains controversial. While some studies report no significant association between embryo storage time and pregnancy or neonatal outcomes (10, 11), others suggest that prolonged vitrification may adverse effect these outcomes (12, 13). Current evidence on embryo cryopreservation is largely restricted to storage durations of <5 years, with studies investigating longer periods limited by small sample sizes. Furthermore, the existing literature is confounded by heterogeneous study populations (mixed cleavage-stage embryos and blastocysts) as well as the lack of consideration of embryo quality (12–16). Consequently, it remains unclear whether extended cryopreservation adversely impacts pregnancy or neonatal outcomes in frozen embryo transfer (FET) cycles.

Therefore, the aim of this study is to investigate whether prolonged embryo cryopreservation duration (> 5 years) influences pregnancy and neonatal outcomes in women undergoing first FET cycles with high-quality blastocyst transfers by using propensity score matching (PSM) analysis.

## 2 Methods

### 2.1 Study design and participants

This multicenter retrospective study included women who underwent single high-quality blastocyst (grade was ≥ 3BB using the Gardner scoring system) transfer during their first FET cycles performed at the reproductive center of Northwest Women’s and Children’s Hospital, the First Affiliated Hospital of Xiamen University, and Tongji Hospital, Tongji Medical College of Huazhong University of Science and Technology from January 2016 and June 2023. Cycles were excluded if they met any of the following criteria: (1) PGT; (2) oocyte donation; (3) uterine malformations, submucosal myomas, or endometrial polyps; (4) incomplete data due to loss to follow-up; (5) non-viable blastocysts after thawing; (6) Anatomically unpassable cervical canal; (7) Cycle cancellation due to acute pre-transfer complications. Furthermore, if multiple cycles for the same woman were recorded in the database, only the first cycle was selected for analysis. Ultimately, a total of 24,101 women were included in this study and categorized into two groups based on the duration of embryo cryopreservation: the short Cryo group consisted of 23,933 women with a storage time of 0–5 years, while the long Cryo group included 168 women with a storage time > 5 years. This study was approved by the Ethics Committee of the Northwest Women’s and Children’s Hospital (number 2023003), Tongji Hospital, Tongji Medical College, Huazhong University of Science and Technology (number 202409024), and the First Affiliated Hospital of Xiamen University (number 20240146). Due to the retrospective nature of this study, the requirement for written informed consent was waived.

### 2.2 Ovarian stimulation and embryo culture

Several conventional controlled ovarian hyperstimulation (COH) protocols were individually determined according to the patient’s age and ovarian reserve indicators (antral follicle count, follicle stimulating hormone, and anti-mullerian hormone). These protocols included short and long gonadotropin-releasing hormone (GnRH) agonists, antagonists, and mild stimulation protocols. Transvaginal oocyte retrieval was carried out 36 hours after the administration of human chorionic gonadotropin (HCG). Subsequently, either conventional *in-vitro* fertilization (IVF) or intracytoplasmic sperm injection (ICSI) was performed, depending on the semen quality and fertilization history. The fertilized oocytes were then cultivated in G1-plus medium (Vitrolife, Sweden) until they reached the cleavage stage (Day 3), and thereafter continuously cultured in G2-plus medium (Vitrolife, Sweden) until they developed into the blastocyst stage (Day 5 or Day 6). The morphology of blastocysts was evaluated using the Gardner scoring system, which is based on the degree of blastocoel expansion (ranging from 1-6), as well as the quality of the inner cell mass (graded as A, B, or C) and trophectoderm (graded as A, B, or C) (17).

### 2.3 Vitrification and warming

The vitrification and warming procedures were carried out with a vitrification kit (Kitazato Company, Japan), in accordance with the protocol detailed in Shi et al. (18). For vitrification, blastocysts were first exposed to equilibration solution (ES) (Kitazato, Japan) for 15 minutes. Subsequently, they were transferred into a vitrification solution (VS) (Kitazato, Japan) for 1 minute. Finally, blastocysts were carefully loaded onto the surface of the Cryotop strip (Kitazato Company, Japan) and immediately immersed in liquid nitrogen at -196 °C for long-term storage. All procedures were conducted at room temperature. During the thawing process, the Cryotop strip was removed from liquid nitrogen and immediately immersed in thawing solution (TS) (Kitazato, Japan) for 1 minute at 37 °C. The embryos were then transferred into dilution solution (DS) (Kitazato, Japan) for 3 minutes, followed by washing in washing solution (WS) (Kitazato, Japan) for 5 minutes at room temperature. Finally, the blastocysts underwent an additional wash in WS (Kitazato, Japan) for 1 minute at 37 °C. The embryos were then placed in G2-Plus medium and cultured until transfer.

### 2.4 Endometrial preparation

Endometrial preparation included natural cycle, hormone replacement therapy (HRT), and GnRH agonist combined with HRT (GnRH agonist-HRT). In general, a natural cycle was used for women with regular menstrual cycles, while HRT or GnRH agonist combined with HRT (GnRH agonist-HRT) was used for women with irregular menstrual cycles. In women with natural cycles, follicular development and ovulation were monitored by transvaginal ultrasound and serum luteinizing hormone (LH) levels. On the 5th day after ovulation, blastocysts were warmed and transferred. For women undergoing HRT or GnRH agonist-HRT, endometrial preparation was initiated with daily oral administration of estradiol valerate (Progynova; Bayer, Berlin, Germany), starting on Day 5 of menstruation. Once the endometrial thickness (EMT) exceeded 7 mm and the serum progesterone level was < 1.5 ng/mL, progesterone was administered via intramuscular injection for corpus luteum support. Blastocysts were then transferred on the 6th day later. Luteal phase support was continued until 10 weeks of gestation in the event of a successful pregnancy. Proficient nurses diligently monitored pregnancy and neonatal outcomes, ensuring they were accurately recorded in the electronic medical record system (19).

### 2.5 Definitions of study outcomes

The primary outcome of our study was live birth, while secondary outcomes included biochemical pregnancy, clinical pregnancy, ectopic pregnancy, miscarriage, and neonatal outcomes. A live birth was defined as the delivery of a live baby after 24 completed weeks of gestation. Biochemical pregnancy was defined as a serum HCG level greater than 20 IU/L on the 12th day after blastocyst transfer. Clinical pregnancy was defined as the presence of at least one gestational sac with fetal heartbeat on transvaginal ultrasound approximately 42 days after embryo transfer. Ectopic pregnancy was diagnosed when a gestational sac was found outside the uterine cavity by ultrasound. Miscarriage was defined as the loss of fetal cardiac activity within 24 weeks of gestation after clinical confirmation (20). The neonatal outcomes included multiple live births, gestational age, birth weight, newborn gender, preterm birth (gestation < 37 weeks), low birthweight (birthweight < 2500 g), and high birth weight (birthweight > 4000 g).

### 2.6 Statistical analysis

All statistical analyses were conducted using SPSS software version 22.0 (SPSS Inc., Chicago, USA) and the statistical packages R (v.3.4.3; The R Foundation). Continuous variables were expressed as the mean and standard deviation (SD), while categorical data were shown as frequencies and percentages. For continuous variables, differences between groups were compared using Student’s t-test if normally distributed; Otherwise, the Mann-Whitney U test was used. Categorical data were compared using the Chi-squared test or Fisher’s exact test, as appropriate. A P-value < 0.05 was considered statistically significant.

The baseline characteristics of the short Cryo group (0–5 years, n = 23,933) and the long Cryo group (> 5 years, n = 168) showed statistically significant differences in maternal age, maternal body mass index (BMI), infertility duration, infertility cause, as well as endometrial preparation protocols. Therefore, PSM without replacement was conducted between the two groups with a caliper width of 0.1 standard deviation and a 1:4 ratio (21). The matching covariates included maternal age, maternal BMI, infertility duration, fertilization type, infertility cause, endometrial preparation protocols, EMT, and infertility type.

## 3 Results

### 3.1 Participant characteristics

**Figure 1:**
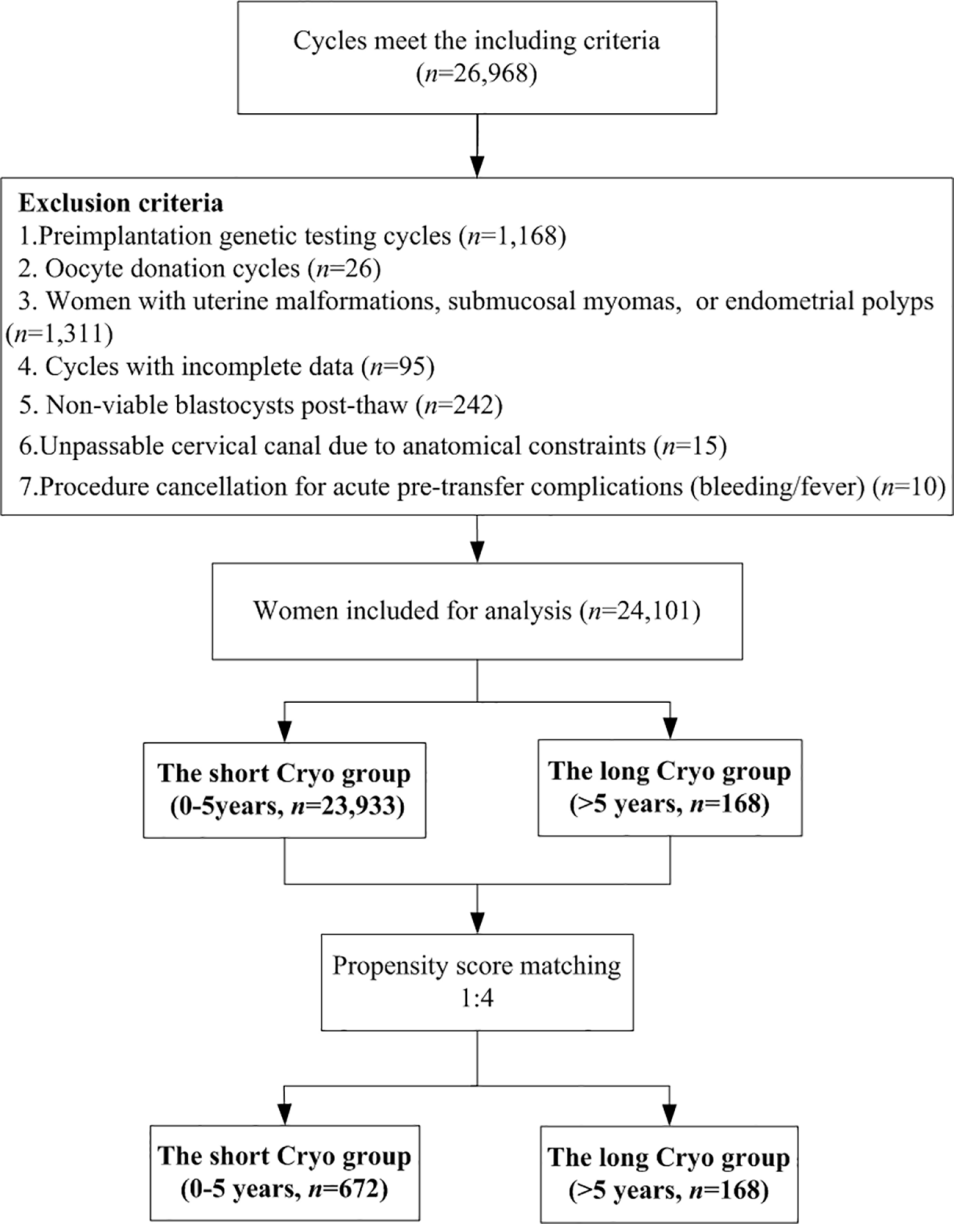
Flow chart of the study.

A total of 24,101 women were included in this study and categorized into two groups according to the duration of embryo cryopreservation: the short Cryo group consisted of 23,933 women with a storage time of 0–5 years, while the long Cryo group included 168 women with a storage time > 5 years. To balance the baseline characteristics, PSM was used to match 168 women in the long Cryo group with 672 women in the short Cryo group (**Figure 1**). The baseline characteristics of the patients were presented in **Table 1**. After matching, no differences were observed between the two groups in terms of maternal age, maternal BMI, infertility duration, fertilization type, infertility cause, endometrial preparation protocols, EMT, and infertility type. In addition, blastocyst survival rates were comparable between short Cryo group and long Cryo group both before and after PSM (P>0.05, for all). Furthermore, study participants were stratified into five groups according to blastocyst cryopreservation duration: 0–2 years, 2–3 years, 3–4 years, 4–5 years, and >5 years. As detailed in **Supplementary Table 1**, pregnancy and neonatal outcomes were generally comparable across all groups, except for gestational age showed statistically significant difference (P<0.001).

**Table 1:** Baseline characteristics of women undergoing single high-quality blastocyst transfer before and after PSM.

Characteristics	Before PSM			After PSM		
	The short Cryo group (0–5 years)	The long Cryo group (>5 years)	*P value*	The short Cryo group (0–5 years)	The long Cryo group (>5 years)	*P value*
Number of cycles, n	23,933	168		672	168	
Maternal age (y)	31.30 ± 4.00	34.00 ± 2.95	<0.001	33.89 ± 3.91	34.00 ± 2.95	0.293
Maternal BMI (kg/m2)	22.21 ± 3.24	21.69 ± 3.13	0.024	21.56 ± 2.76	21.69 ± 3.13	0.907
Infertility duration (y)	3.25 ± 2.31	3.88 ± 2.82	0.01	3.86 ± 2.71	3.88 ± 2.82	0.855
EMT (mm)	9.90 ± 1.75	10.00 ± 2.03	0.161	10.09 ± 1.81	10.00 ± 2.03	0.932
Infertility type, n (%)			0.647			0.971
Primary	14,688 (61.37)	106 (63.10)		425 (63.24)	106 (63.10)	
Second	9,245 (38.63)	62 (36.90)		247 (36.76)	62 (36.90)	
Infertility cause, n (%)			<0.001			0.874
Male	4,664 (19.49)	35 (20.83)		128 (19.05)	35 (20.83)	
Female	16,050 (67.06)	94 (55.95)		393 (58.48)	94 (55.95)	
Both	1,724 (7.20)	30 (17.86)		122 (18.15)	30 (17.86)	
Unkown	1,495 (6.25)	9 (5.36)		29 (4.32)	9 (5.36)	
Endometrial preparation protocols, n (%)			<0.001			0.595
Natural cycle	3,482 (14.55)	52 (30.95)		189 (28.12)	52 (30.95)	
HRT	16,301 (68.11)	93 (55.36)		401 (59.67)	93 (55.36)	
GnRH agonist-HRT	4,150 (17.34)	23 (13.69)		82 (12.20)	23 (13.69)	
Fertilization type, n (%)			0.195			0.555
IVF	18,359 (76.71)	136 (80.95)		557 (82.89)	136 (80.95)	
ICSI	5,574 (23.29)	32 (19.05)		115 (17.11)	32 (19.05)	
Survival rate (%)			0.711			0.853
Yes	23,933 (99.01)	168 (98.82))		672 (98.97)	168 (98.82)	
No	240 (0.99)	2 (1.18)		7 (1.03)	2 (1.198)	

Values are presented as mean ± standard deviation or n (%).

PSM, propensity matching; BMI, body mass index; EMT, endometrial thickness; HRT, hormone replacement therapy; GnRH, gonadotropin-releasing hormone; IVF, in vitro fertilization; ICSI, intracytoplasmic sperm injection.

### 3.2 Pregnancy and neonatal outcomes

The pregnancy and neonatal outcomes were summarized in **Table 2**. The pregnancy outcomes were comparable between the two groups, including biochemical pregnancy, clinical pregnancy, ectopic pregnancy, miscarriage, live birth, and multiple live births (P > 0.05 for all). The neonatal outcomes of singleton infants were also evaluated. Compared to the short Cryo group, the gestational age in the long Cryo group was slightly reduced (P < 0.05). However, no differences were observed between the two groups in other neonatal outcomes in terms of birth weight, cesarean delivery, newborn gender, very preterm birth, preterm birth, very low birth weight, low birth weight, and high birth weight (P > 0.05, for all). Notably, the results were similar both before and after PSM.

**Table 2:** Pregnancy and neonatal outcomes of women undergoing single high-quality blastocyst transfer before and after PSM.

Outcomes	Brfore PSM			After PSM		
	The short Cryo group (0–5 years)	The long Cryo group (>5 years)	*P value*	The short Cryo group (0–5 years)	The long Cryo group (>5 years)	*P value*
Biochemical pregnancy, n (%)	17,187 (71.81)	121 (72.02)	0.952	479 (71.28)	121 (72.02)	0.849
Clinical pregnancy, n (%)	15,654 (65.41)	112 (66.67)	0.732	435 (64.73)	112 (66.67)	0.638
Ectopic pregnancy, n (%)	103 (0.43)	2 (1.19)	0.136	4 (0.60)	2 (1.19)	0.345
Miscarriage, n (%)	2,639 (11.03)	17 (10.12)	0.708	70 (10.42)	17 (10.12)	0.91
Live birth, n (%)	12,915 (53.96)	93 (55.36)	0.718	361 (53.72)	93 (55.36)	0.703
Multiple live birth, n (%)	223 (1.73)	2 (2.15)	0.755	9 (2.49)	2 (2.15)	0.848
Cesarean delivery, n (%)	2,722 (21.08)	15 (16.13)	0.243	75 (20.78)	15 (16.13)	0.316
Preterm birth, n (%)	1,093 (8.61)	8 (8.79)	0.952	25 (7.10)	8 (8.79)	0.584
Very preterm birth, n (%)	132 (1.04)	0 (0.00)	0.328	4 (1.14)	0 (0.00)	0.586
Male gender, n (%)	7,343 (56.86)	55 (59.14)	0.658	212 (58.73)	55 (59.14)	0.942
Very low birth weight (<1,500 g), n (%)	93 (0.73)	0 (0.00)	0.412	5 (1.42)	0 (0.00)	0.588
Low birth weight (<2,500 g), n (%)	620 (4.88)	2 (2.20)	0.235	14 (3.98)	2 (2.20)	0.417
High birth weight (>4,000 g), n (%)	757 (5.96)	7 (7.69)	0.488	12 (3.41)	7 (7.69)	0.072
Birth weight (g)	3,304.86 ± 522.85	3,336.40 ± 457.70	0.78	3,309.64 ± 511.79	3,336.40 ± 457.70	0.959
Gestational age (week)	38.67 ± 1.77	38.44 ± 1.33	0.004	38.70 ± 1.81	38.44 ± 1.33	0.007

Values are presented as mean ± standard deviation or n (%).

PSM, propensity matching.

To further explore the influence of cryopreservation duration on pregnancy and neonatal outcomes, a multivariate logistic regression analysis was performed to adjust for potential confounding factors, including maternal age, maternal BMI, infertility duration, fertilization type, infertility cause, endometrial preparation protocols, EMT, and infertility type (**Tables 3**, **4**). After adjusting for potential confounding factors, no significant differences were observed between the two groups in pregnancy outcomes, including biochemical pregnancy (adjust odds ratio [aOR] 1.04, 95% confidence interval [CI], 0.70–1.56; P = 0.831), clinical pregnancy (aOR 1.10, 95% CI, 0.75–1.60; P = 0.638), ectopic pregnancy (aOR 2.15, 95% CI, 0.37–12.56; P = 0.394), miscarriage (aOR 0.95, 95% CI, 0.54–1.69; P = 0.871), and live birth (aOR 1.09, 95% CI, 0.76–1.55; P = 0.646). In addition, no significant differences were observed in neonatal outcomes, including very preterm birth, preterm birth, very low birth weight, low birth weight, high birth weight, birth weight, and gestational age (P > 0.05, for all).

**Table 3:** Odds ratios of pregnancy outcomes and neonatal outcomes in different models.

	Crude model^a^		Adjusted model I^b^		Adjusted model II^c^	
	OR (95% CI)	*P value*	aOR (95% CI)	*P value*	aOR (95% CI)	*P value*
Biochemical pregnancy	1.04 (0.71, 1.51)	0.849	1.00 (0.68, 1.47)	0.993	1.04 (0.70, 1.56)	0.831
Clinical pregnancy	1.09 (0.76, 1.56)	0.638	1.05 (0.73, 1.53)	0.783	1.10 (0.75, 1.60)	0.638
Ectopic pregnancy	2.01 (0.37, 11.08)	0.422	2.21 (0.40, 12.31)	0.367	2.15 (0.37, 12.56)	0.394
Miscarriage	0.97 (0.55, 1.69)	0.91	0.95 (0.54, 1.68)	0.861	0.95 (0.54, 1.69)	0.871
Live birth	1.07 (0.76, 1.50)	0.703	1.05 (0.74, 1.49)	0.778	1.09 (0.76, 1.55)	0.646
Multiple live birth	0.86 (0.18, 4.05)	0.848	1.13 (0.23, 5.54)	0.88	1.14 (0.21, 6.24)	0.876
Very preterm birth	0.00 (0.00, Inf)	0.993	0.00 (0.00, Inf)	1	0.00 (0.00, Inf)	1
Preterm birth	1.26 (0.55, 2.90)	0.585	1.13 (0.49, 2.64)	0.772	1.20 (0.50, 2.90)	0.68
Very low birth weight	0.00 (0.00, Inf)	0.993	0.00 (0.00, Inf)	1	0.00 (0.00, Inf)	1
Low birth weight	0.54 (0.12, 2.43)	0.424	0.53 (0.12, 2.37)	0.402	0.53 (0.11, 2.54)	0.43
High birth weight	2.36 (0.90, 6.18)	0.08	2.24 (0.84, 5.96)	0.108	2.45 (0.89, 6.78)	0.084

a No adjustments for other covariates.

b Adjusted for maternal age and BMI.

c Adjusted for maternal age; maternal BMI; infertility duration; fertilization type; infertility cause; endometrial preparation protocols; EMT; infertility type.

OR, odds ratio; CI, confidence interval; aOR, adjust odds ratio; BMI, body mass index; EMT, endometrial thickness.

**Table 4:** Multivariable linear regression of gestational age and birth weight.

		B	Standard error	P value
Birth weight	Crude model^a^	26.758	58.289	0.646
	Adjusted model I^b^	22.76	58.592	0.698
	Adjusted model II^c^	23.101	58.834	0.695
Gestational age	Crude model^a^	-0.256	0.2	0.201
	Adjusted model I^b^	-0.21	0.2	0.294
	Adjusted model II^c^	-0.214	0.2	0.286

a No adjustments for other covariates.

b Adjusted for maternal age and BMI.

c Adjusted for maternal age; maternal BMI; infertility duration; fertilization type; infertility cause; endometrial preparation protocols; EMT; infertility type.

## 4 Discussion

With the increased application of cryopreserved embryos through vitrification, there has been ongoing debate regarding whether the duration of vitrification affects pregnancy and neonatal outcomes. This large, multicenter study included 24,101 women who underwent single high-quality blastocyst transfer and demonstrated that Prolonged cryopreservation duration has no significant association on pregnancy or neonatal outcomes.

Although the topic remains debatable, the majority of studies have reported no significant effect of vitrification duration on pregnancy and neonatal outcomes. A large retrospective study involving 58,001 women, including both single and double embryo transfers, found no significant association between vitrification duration and clinical outcomes (11). Furthermore, studies by Cimadomo et al. and Sekhon et al. demonstrated that vitrification durations of up to 3 years and 4 years, respectively, have no adverse effect on pregnancy and neonatal outcomes (22, 23). A recent study involving women undergoing either cleavage or blastocyst-stage transfers also showed that vitrification durations did not impair pregnancy or neonatal outcomes (16). Consistent with previous studies, this multicenter retrospective analysis found no evidence of significant associations between prolonged cryopreservation of high-quality blastocysts and adverse pregnancy or neonatal outcomes.

Meanwhile, two recent studies have reported that prolonged cryopreservation negatively impacts biochemical pregnancy rates, clinical pregnancy rates, and live birth rates. A retrospective study conducted by Li et al., based on 24,698 women, demonstrated that the rates of biochemical pregnancy, clinical pregnancy, and live birth decreased with prolonged storage time; however, it did not influence neonatal outcomes (12). It is noteworthy that 92.8% of the embryos transferred were at the cleavage stage. Surprisingly, both the clinical pregnancy rate and live birth rate were significantly decreased after just 3 months of cryopreservation, which should be treated with caution as it challenges the principles of cryobiology. Parmegiani and Vajta have expressed skepticism regarding that storage time is solely responsible for the declining success rates observed in this study. They propose that factors such as maternal age, embryo characteristics (e.g., quality, stage, number), and cryo-storage conditions may also contribute to the observed outcomes (24). Another recent study conducted by Yan Y et al., involving a total of 6,900 women who underwent blastocyst transfer, demonstrated that long-term blastocyst vitrification for more than 6 years negatively affects biochemical pregnancy, clinical pregnancy, and live birth rates, but does not impact neonatal outcomes (25). However, women with a storage duration exceeding 6 years showed an increase in maternal age, along with a reduced number of transferred blastocysts, a lower proportion of high-quality blastocysts, and thinner EMT. Therefore, it remains unclear whether the reduced rates of biochemical pregnancy, clinical pregnancy, and live birth were due to long-term cryopreservation.

A study by Pruksananonda K et al. demonstrated that human embryos retained their pluripotency, similar to fresh embryos, even after 18 years of cryopreservation (26). Evidence suggests that when temperatures drop below -130 °C, many cells can remain stable for extended periods, as enzyme activity is nearly completely suppressed in liquid nitrogen (27). The only study to examine DNA integrity in human embryos indicated that vitrification has a much lesser impact on DNA integrity compared to slow freezing (28). Furthermore, a recent study demonstrated that vitrified storage time did not affect mRNA and lncRNA expression profiles in human embryos; however, the vitrification warming procedure could lead to minor alteration in transcriptome (29). Therefore, during long-term storage, any potential adverse effects on pregnancy and neonatal outcomes may not primarily be attributed to the duration of cryopreservation, but rather to the storage conditions and the procedures performed by embryologists (8). In this study, liquid nitrogen was replenished weekly, and electronic systems for monitoring the temperature of liquid nitrogen were in place. Neither the applied protocol nor the vitrification methodology was altered throughout the study period. Additionally, the embryologists have undergone strict training in vitrification techniques and were periodically evaluated as part of the quality control process in our cryo-laboratories. In this study, blastocyst survival rates were comparable between short Cryo group and long Cryo group both before and after PSM. Thus, the present study provides convincing evidence regarding the effect of long-term cryopreservation on pregnancy and neonatal outcomes.

Notably, this study has several strengths. Firstly, it draws robust conclusions based on a large-scale dataset obtained from three tertiary academic medical centers. Secondly, PSM was employed to reduce potential confounding effects on pregnancy and neonatal outcomes. Thirdly, the categorization of samples was not arbitrary; women included in this study were classified into two groups based on the controversial 5-year vitrification duration threshold, as highlighted in several previously published studies (13, 15, 25). An additional strength of this study is the remarkable stability of clinical protocols throughout the study period, including consistent laboratory procedures, endometrial preparation regimens, and embryo transfer techniques. This stability minimizes potential temporal confounding and strengthens the validity of the results of this study.

Nonetheless, this study has several limitations. The primary limitation is that there are only 13 women with storage durations > 8 years; therefore, caution should be exercised when interpreting the results. Additionally, despite the implementation of rigorous inclusion and exclusion criteria, the retrospective nature of the study may have introduced selection bias. To partially mitigate this, we limited our analysis to women who underwent the transfer of a single high-quality blastocyst. Furthermore, the lack of long-term follow-up on offspring restricts our ability to provide more definitive evidence regarding the potential effects of vitrification duration on offspring health. Last but not least, although our observational study found no significant association between cryopreservation duration and clinical outcomes, the absence of *a priori* power calculations and reproductive history data limit definitive interpretation of these results. Therefore, future prospective studies with adequately powered designs are warranted to confirm these findings.

## 5 Conclusion

In conclusion, this multicenter retrospective analysis found no evidence of significant associations between prolonged cryopreservation of high-quality blastocysts and adverse pregnancy or neonatal outcomes. This provides strong evidence for the safety of embryo vitrification, even after extended storage periods, offering reassurance to patients undergoing FET cycles.

## Data Availability

The original contributions presented in the study are included in the article/**Supplementary Material**. Further inquiries can be directed to the corresponding authors.
