# Supplementary material for: Cryopreservation duration does not affect pregnancy or neonatal outcomes in single high-quality blastocyst transfers: a multicenter retrospective study
Source: Front Endocrinol (Lausanne). 2026 Mar 6;17:1743765. doi: 10.3389/fendo.2026.1743765 (PMC13002392; doi:10.3389/fendo.2026.1743765)
Supplement: Supplementary file 1 [file Table1.docx]

**Supplementary Table 1** Detailed analysis of pregnancy and neonatal outcomes stratified by blastocyst cryopreservation duration

| **Outcomes** | **Group 1 (0-2)** | **Group 2 (2-3)** | **Group 3 (3-4)** | **Group 4 (4-5)** | **Group 5 (>5)** | **P value** |
| --- | --- | --- | --- | --- | --- | --- |
| Biochemical pregnancy, n (%) | 16,207 (71.84) | 425 (71.91) | 353 (71.46) | 202 (70.38) | 121 (72.02) | 0.988 |
| Clinical pregnancy, n (%) | 14,745 (65.36) | 393 (66.50) | 327 (66.19) | 189 (65.85) | 112 (66.67) | 0.961 |
| Ectopic pregnancy, n (%) | 96 (0.43) | 2 (0.34) | 1 (0.20) | 4 (1.39) | 2 (1.19) | 0.059 |
| Miscarriage, n (%) | 2,484 (11.01) | 69 (11.68) | 56 (11.34) | 30 (10.45) | 17 (10.12) | 0.969 |
| Live birth, n (%) | 12,168 (53.93) | 322 (54.48) | 270 (54.66) | 155 (54.01) | 93 (55.36) | 0.99 |
| Multiple live births, n(%) | 210 (1.73) | 7 (2.17) | 5 (1.85) | 1 (0.65) | 2 (2.15) | 0.87 |
| Preterm birth, n (%) | 1,019 (8.54) | 25 (7.94) | 37 (13.96) | 12 (7.79) | 8 (8.79) | 0.051 |
| Male gender, n (%) | 6,924 (56.90) | 186 (57.76) | 140 (51.85) | 93 (60) | 55 (59.14) | 0.45 |
| Low birth weight (<2,500 g), n (%) | 581 (4.86) | 15 | 14 | 10 | 2 (2.20) | 0.67 |
| High birth weight (>4,000 g), n (%) | 701 (5.86) | 26 | 28 | 12 | 7 (7.69) | 0.30 |
| Gestational age (week) | 38.69±1.78 | 38.51 ± 1.65 | 38.25 ± 1.65 | 38.42 ± 1.75 | 38.44±1.33 | <0.001 |
| Birth weight (g) | 3,303.67±521.51 | 3,324.28 ± 543.93 | 3,316.01 ± 539.50 | 3,338.68 ± 555.80 | 3,336.40±457.70 | 0.81 |
